# Good practices for the automated production of ^18^F-SiFA radiopharmaceuticals

**DOI:** 10.1186/s41181-023-00215-1

**Published:** 2023-10-11

**Authors:** Simon Blok, Carmen Wängler, Peter Bartenstein, Klaus Jurkschat, Ralf Schirrmacher, Simon Lindner

**Affiliations:** 1 Department of Nuclear Medicine and Molecular Imaging, University Medical Center Groningen, University of Groningen, Groningen, The Netherlands; 2 Biomedical Chemistry, Clinic of Radiology and Nuclear Medicine, Medical Faculty Mannheim, Heidelberg University, Mannheim, Germany; 3 Department of Nuclear Medicine, LMU University Hospital, LMU Munich, Munich, Germany; 4 Fakultät für Chemie und Chemische Biologie, Technische Universität Dortmund, Dortmund, Germany; 5 Department of Oncology, Division of Oncological Imaging, University of Alberta, Edmonton, AB Canada

**Keywords:** Fluorine-18, Silicon fluoride acceptor, Automation, Positron emission tomography (PET)

## Abstract

**Background:**

The positron emitting isotope fluorine-18 (^18^F) possesses almost ideal physicochemical properties for the development of radiotracers for diagnostic molecular imaging employing positron emission tomography (PET). ^18^F in its nucleophilic anionic ^18^F^−^ form is usually prepared by bombarding an enriched ^18^O water target with protons of various energies between 5 and 20 MeV depending on the technical specifications of the cyclotron. Large thick-target yields between 5 and 14 GBq/µA can be obtained, enough to prepare large batches of radiotracers capable to serve a considerable contingent of patients (50 + per clinical batch). The overall yield of the radiotracer however depends on the efficiency of the ^18^F labeling chemistry. The Silicon Fluoride Acceptor chemistry (SiFA) has introduced a convenient and highly efficient way to provide clinical peptide-based ^18^F-radiotracers in a kit-like procedure matching the convenience of ^99m^Tc radiopharmaceuticals.

**Main body:**

A radiotracer’s clinical success primarily hinges on whether its synthesis can be automated. Due to its simplicity, the SiFA chemistry, which is based on isotopic exchange (^18^F for ^19^F), does not only work in a manual setup but has been proven to be automatable, yielding large batches of ^18^F-radiotracers of high molar activity (A_m_). The production of SiFA radiotracer can be centralized and the radiopharmaceutical be distributed via the “satellite” principle, where one production facility economically serves multiple clinical application sites. Clinically validated tracers such as [^18^F]SiTATE and [^18^F]Ga-rhPSMA-7/-7.3 have been synthesized in an automated synthesis unit under good manufacturing practice conditions and used in large patient cohorts. Communication of common guidelines and practices is warranted to further the dissemination of SiFA radiopharmaceuticals and to give easy access to this technology.

**Conclusion:**

This current review highlights the most recent achievements in SiFA radiopharmaceutical automation geared towards large batch production for clinical application. Best practice advice and guidance towards a facilitated implementation of the SiFA technology into new and already operating PET tracer production facilities is provided. A brief outlook spotlights the future potential of SiFA radiochemistry within the landscape of non-canonical labeling chemistries.

## Background

**Fig. 1:**
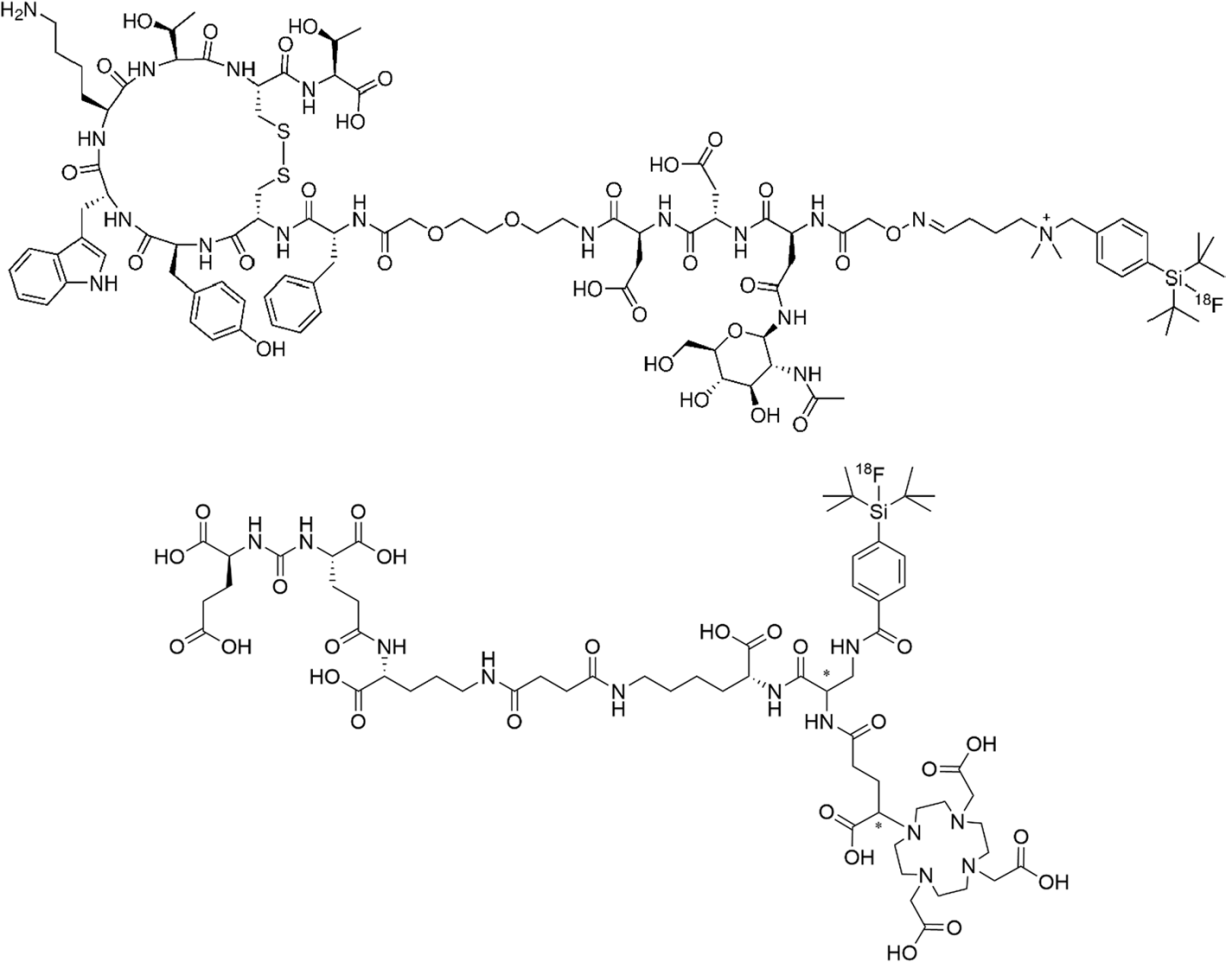
Structures of [^18^F]SiTATE (top) and [^18^F]rhPSMA-7 (below, * diastereomeric mixture)

Despite the recent advent and success of novel radionuclides for clinical PET imaging such as gallium-68 (Nelson et al. 2022), zirconium-89, copper-64 and various scandium radioisotopes (^44^Sc and ^43^Sc) (Lima et al. 2021; Qaim and Spahn 2018), ^18^F still consolidates its top position as a clinically relevant nuclide for PET tracer development. Its simple and efficient production via the high-yield ^18^O(p,n)^18^F nuclear reaction, the simple yet highly efficient radiochemistries available to perform high-yield labeling reactions of even complex precursors, and ^18^F’s almost ideal physicochemical properties, such as clinically suitable half-life (t_½_ = 110 min), a low positron energy (0.635 MeV, maximum range in water = 2.4 mm) and high positron emission ratio (97%), make ^18^F the most frequently applied radionuclide in clinical diagnostic PET imaging. Fluorine has a small von der Waals radius of 1.47 Å, close to that of hydrogen (1.2 Å) and can therefore replace hydrogen in any molecule without dramatically altering its molecular dimensions. The fluorine-carbon bond strength (112 kcal/mol) is very high despite fluorine’s high electronegativity and resists oxidation and heat exposure, ideal prerequisites for the often harsh conditions used in PET tracer synthesis. ^18^F can be produced in large quantities of many Giga-Becquerels allowing for the production of large clinical radiotracer batches delivering clinical doses serving up to 50 patients and more. Utilizing proton energies between 5 and 20 MeV for the bombardment of ^18^O-water targets, thick-target yields of 5–14 GBq/µA are possible. Furthermore, the large number of installed cyclotrons worldwide ensures stable supply lines and guarantees a widespread availability. The half-life of ^18^F enables a centralized production, which can serve as a supply hub for many clinical facilities within a radius of many hundreds of kilometers (“satellite principle”). Despite all these positive features, common radiolabeling protocols with ^18^F can be complex and difficult to automate. Especially compounds of higher molecular weight such as peptides cannot be easily labeled in one-step at higher temperatures, normally required to form a carbon–fluorine bond in situ. Radiolabeled peptides however have become an important and integral part of modern clinical imaging, especially in the field of oncologic diagnostic PET (Jackson et al. 2017). Non canonical ^18^F-labeling approaches introducing aluminium-^18^F (Archibald and Allott 2021), boron-^18^F (Bernard-Gauthier et al. 2016), sulfur-^18^F (Zheng et al. 2021) and silicon-^18^F chemistries (Gower-Fry et al. 2021), have gradually replaced commonly applied prosthetic group labeling protocols for peptides, introducing a new era of ^18^F-PET tracer development. Most of these chemistries have been successfully consolidated in clinical diagnostic PET imaging and with even more methodologies on the horizon (e.g. Ga-^18^F radiochemistry) (Monzittu et al. 2018), the future prospects of ^18^F usage in PET are rather positive. The silicon fluoride acceptor labeling approach (SiFA) is one among the above-mentioned new labeling chemistries characterized by high radio fluorination yields under generally very mild conditions. The SiFA chemistry was the first radiochemical approach invalidating the common believe that a radiolabeling reaction based on isotopic exchange (IE) only yields compounds of low molar activity (A_m_). SiFA IE as a method to radiolabeling peptides was consequently implemented into B-^18^F and S-^18^F radiolabeling, providing one-step labeling protocols yielding radiotracer of high A_m_ and being devoid of complex workup procedures providing a high level of convenience. The SiFA method lends itself exemplary well to automation. The clinical good manufacturing practice (GMP) conform automated production of two SiFA-based radiotracers, namely [^18^F]SiTATE and [^18^F]Ga-rhPSMA-7/-7.3 (Fig. 1), for imaging tumors of neuroendocrine origin and PSMA levels in prostate cancer respectively and their application in more than thousand clinical cases provides motive to communicate current best practices for their syntheses. These two examples provide a solid base for any future SiFA based radiotracer production since the underlying radiochemistry is straightforward and generally applicable.

## Production of ^18^F

Fluorine-18 can be produced in a medical cyclotron in two different ways: [^18^F]F_2_ is obtained in a gas target by bombardment of either [^18^O]O_2_ with protons via the ^18^O(p,n)^18^F reaction or by deuteron irradiation of Ne via the ^20^Ne(d,α)^18^F pathway. [^18^F]F_2_ is used for electrophilic fluorination reactions. For SiFA chemistry, nucleophilic [^18^F]fluoride is required to displace fluoride-19 with the positron-emitting isotope fluoride-18 via isotopic exchange. Nucleophilic [^18^F]fluoride is produced in a liquid target by proton bombardment of ^18^O-enriched water via the ^18^O(p,n)^18^F reaction to obtain an aqueous solution of [^18^F]fluoride. At the LMU Munich, a GE PETtrace 880™ cyclotron is used which is capable to produce up to 550 GBq fluorine-18 in dual beam mode or 275 GBq in single beam mode with a saturation yield of 6956 MBq/µA. For regular [^18^F]SiTATE syntheses, H^─^-ions are accelerated with a beam current of 65 µA (single beam). After passing the extraction foil, the resulting protons hit the target filling (2.5 g [^18^O]H_2_O) with a beam energy of 16.5 MeV. The irradiation to obtain a starting activity of 60 GBq [^18^F]fluoride takes about 25 min.

## Silicon fluoride acceptor (SiFA) radiochemistry

### Manual SiFA labeling

As a result of its simplicity and technical convenience, the SiFA labeling method is well suited for manual radiolabeling. The SiFA reaction is based on IE and generally characterized by the absence of side product formation. The precursor and ^18^F-labeled product in a SiFA reaction are chemically identical, giving access to an easy workup procedure, where the organic fraction composed of the precursor and labeled product, only have to be separated from inorganic components such as unreacted ^18^F^−^, K_2_CO_3_ and oxalate. This reduces the technical effort of the SiFA reaction to a high yield one-pot radiolabeling reaction devoid of any side product formation. The purification is equally simple. Only a single C18 cartridge extraction/elution of the ^19^F/^18^F SiFA molecule from the solid support is necessary to obtain the small molecule or peptidic SiFA radiotracer dissolved in ethanol. This solution can be easily diluted with saline, buffer (e.g., phosphate buffered saline, PBS) or any other aqueous based injection solution. The manual synthesis of SiFA radiopharmaceuticals including clinically used [^18^F]SiTATE for neuroendocrine tumor imaging, has been extensively reviewed previously (Lindner et al. 2020b). It should be kept in mind that a manual radiosynthesis has different requirements with regard to radiation safety and clinical applicability. To reduce the personnel’s exposure to radiation, a manual synthesis is usually unsuited to satisfy clinical needs and rather serves the purpose of optimizing the radiochemistry needed to establish an automated production of the radiotracer.

### Automated synthesis of [^18^F]SiTATE

**Fig. 2:**
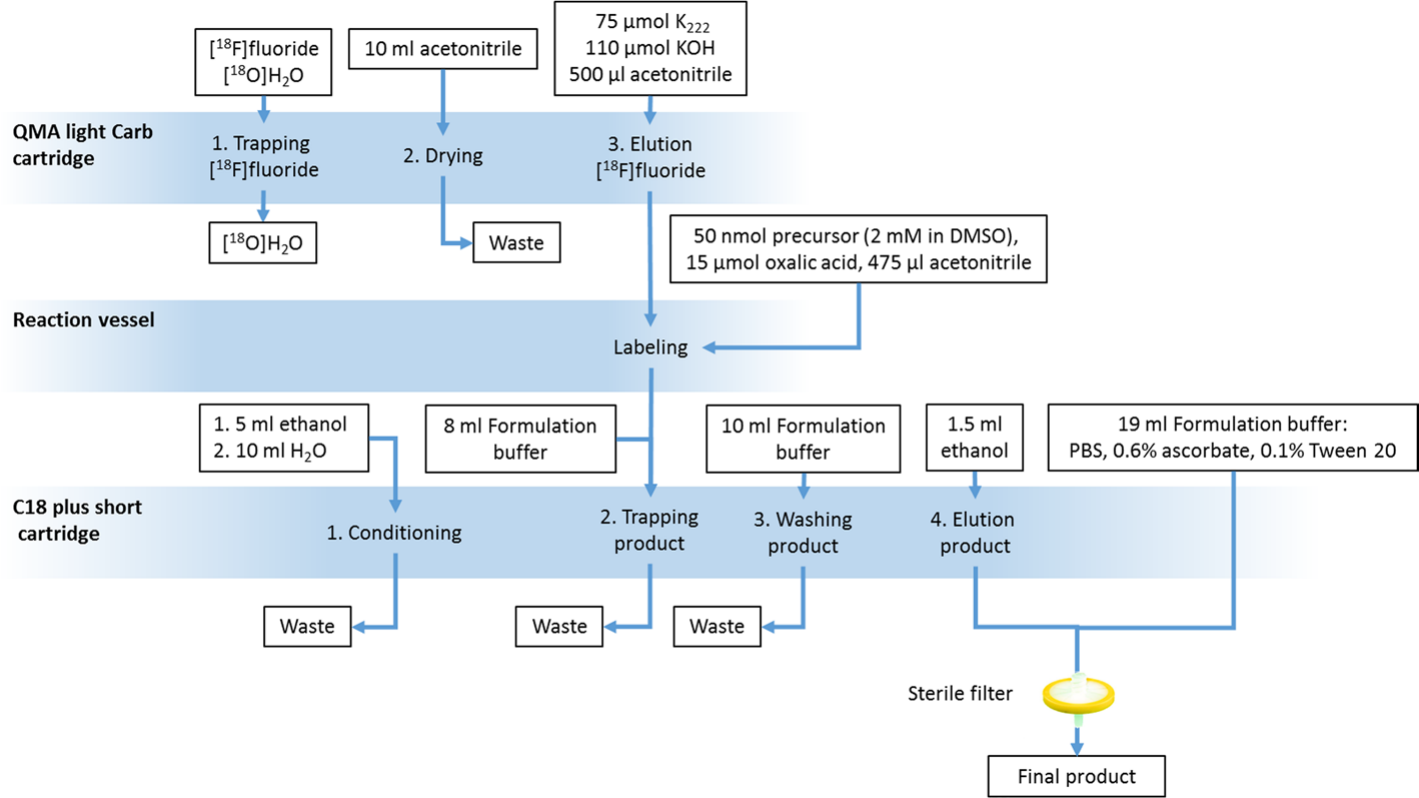
Flow scheme of the [^18^F]SiTATE production on a Scintomics GRP™ 2V ASU

The first automated synthesis of [^18^F]SiTATE for clinical routine production in a GMP environment was reported by Lindner et al. (Lindner et al. 2020a). It was designed as a straightforward approach to provide clinical batches for in-house applications. The process was developed on a Scintomics GRP™ 2V automated synthesis unit (ASU) (Fig. 2). [^18^F]fluoride drying was achieved by a cartridge-based drying method (the so-called Munich method) replacing the conventional more complex and time-consuming azeotropic drying step (Wessmann et al. 2012). The advantage of this method lies in a significant reduction of hardware complexity and time consuming intermediary steps prior to labeling. By this, losses of radioactivity due to radioactive decay and overall complexity are minimized. However, this process is patented and cannot be used for commercialization except by the inventor. The elution cocktail consists of a K_222_/KOH complex (75 µmol K_222_, 110 µmol KOH), which has to be prepared in advance by lyophilisation of respective aqueous solutions to dryness. 50 nmol precursor and 15 µmol oxalic acid, required to neutralize the amount of base, is pre-filled in the reactor before elution. The reaction occurs at room temperature within 5 min without any significant by-product formation. Hence, a simple C18 RP cartridge purification is sufficient to separate residual [^18^F]fluoride, inorganic salts such as KOH, and polar organic components such as oxalate and the cryptand from the product. Acetonitrile is removed by washing the cartridge with aqueous formulation solution. The product is eluted from the cartridge with ethanol and is formulated with PBS, pH 6, supplemented with 0.6% (m/m) ascorbate as stabilizer and 0.1% (m/m) Tween 20 as surfactant. After sterile filtration, the final product is obtained for quality control analyses and clinical application.

308 representative routine productions, carried out at the LMU Munich, were evaluated. Only 6 batches (1.9%) didn´t meet the specifications due to low radiochemical purity and were not released. Starting activities were 63 ± 9 GBq and average radiochemical yields of 53 ± 10% end-of-synthesis corresponding to 62 ± 11% after decay correction were achieved. Molar activities were calculated to be 664 ± 145 GBq/µmol, based on the starting amount of precursor and starting activities of 63 ± 9 GBq.

Meanwhile, [^18^F]SiTATE is also routinely manufactured and implemented in clinical routine at various other sites. At University Medical Center Groningen, an Eckert and Ziegler modular lab system is used. The general reaction pathway is identical, only reagent amounts were adjusted. The elution cocktail contains 100 µmol K_222_ and KOH each, oxalic acid was increased marginally to 17 µmol and 40 nmol precursor is used. The reaction mixture is diluted with 80 mL of water and extracted using a Sep-Pak C18 Plus Light cartridge. After washing the cartridge with 10 mL PBS, the product is eluted using 1 mL ethanol and formulated with 10 mL PBS. The synthesis time is 40 min and thus considerably longer than the LMU protocol with 22 min. The average decay-corrected radiochemical yield is 32 ± 1% (n = 3). Other automation approaches, e.g. on a GE Fastlab ASU, have been envisaged, but data have not been reported yet. But, since the chemistry is simple and straightforward, it should be possible to implement the SiFA methodology practically on any type of automated system.

The success of several clinical studies showing the strong clinical potential for GEP-NET diagnostics and other indications (Beyer et al. 2021; Eschbach et al. 2023; Ilhan et al. 2020, 2019; Unterrainer et al. 2023, 2021) has also put industry partners on the map to develop commercial alternatives including the manufacture of GMP grade starting materials and reagents. ABX has developed a process for the GE TRACERlab MX as well as for the ORA Neptis® RS/DB synthesizer. Both processes rely on the same reaction pathway as described above. The main difference is the integration of azeotropic drying of [^18^F]fluoride in contrast to the cartridge based drying technique. However, this standard process is technically very well established and fully compatible with SiFA chemistry. The total synthesis time is approximately 30 min. GMP grade precursor and materials will be provided to comply with full GMP manufacture standards required to carry out clinical studies.

Also RQS (Ruffani Quality Solutions) provides a novel process to be implemented on the Trasis AllinOne ASU without the need of any HPLC equipment. This process also relies on a 5-min azeotropic drying procedure followed by a 5-min labeling step at room temperature. The purification of the crude solution is achieved using a C18 cartridge. The total synthesis time has been reported to be 35 min with an overall yield of 40 ± 5% (n = 5) end-of-synthesis and a radiochemical purity of ≥ 95%.

### Automated synthesis of [^18^F]Ga-rhPSMA-7/-7.3

**Fig. 3:**
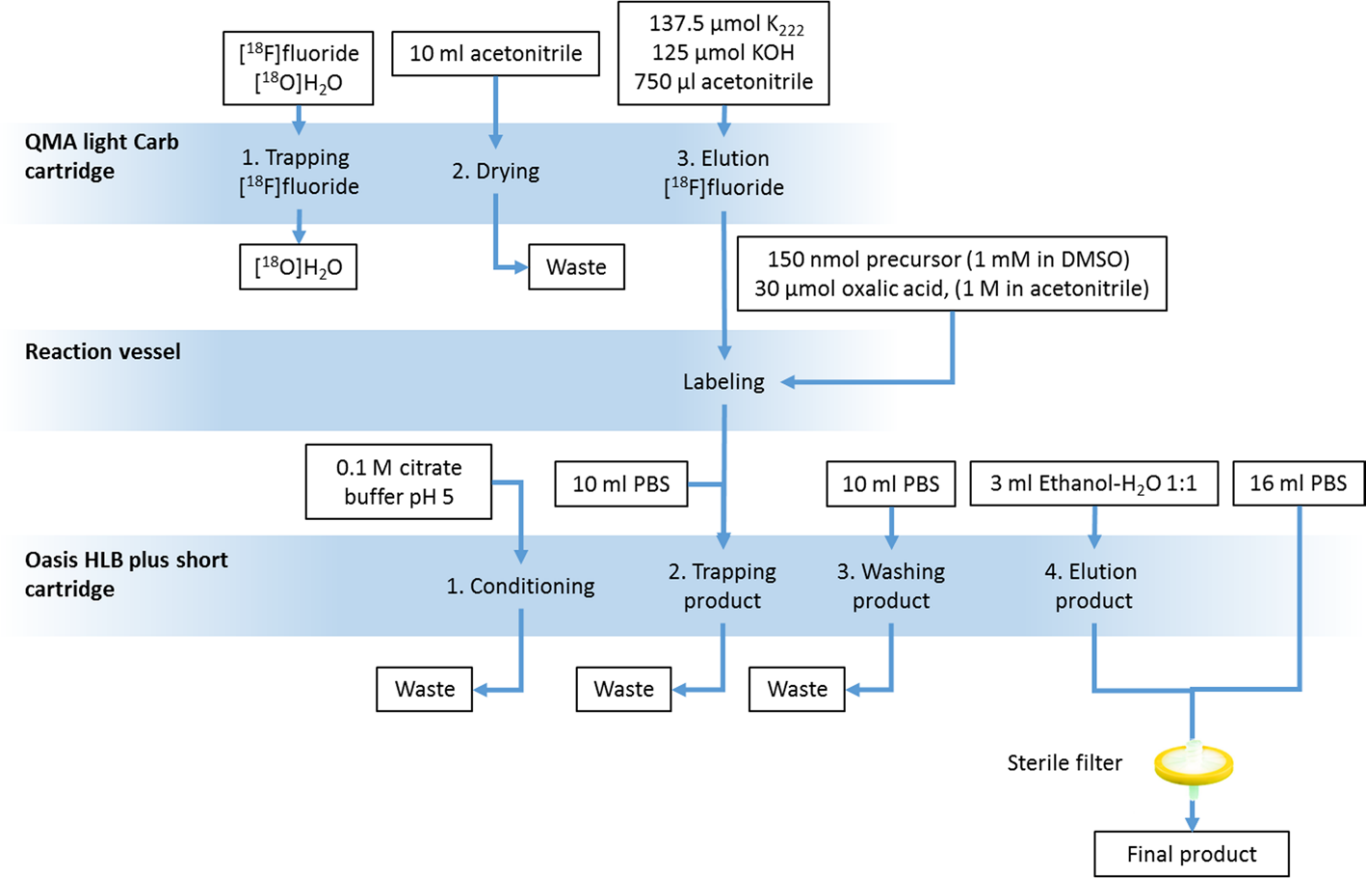
Flow scheme of the [^18^F]Ga-rhPSMA-7/-7.3 production on a Scintomics GRR™ 2V ASU

In 2021, the automation and experience from 243 routine productions of [^18^F]Ga-rhPSMA-7/-7.3 was published (Wurzer et al. 2021). In the case of ^18^F-radiolabeling of the SiFA moiety, the so-called radiohybrid tracer is complexed with non-radioactive gallium and is used in its metalated form as starting material. The synthesis was developed using a Scintomics GRP 2V ASU and strongly resembles the setup used for the [^18^F]SiTATE synthesis (Fig. 3).

In contrast to [^18^F]SiTATE synthesis, three-fold precursor amounts are used ensuring maximal [^18^F]fluoride incorporation. The purification is performed using an Oasis HLB cartridge, which revealed a higher extraction efficiency compared to SepPak C18 materials. The process is very fast and provides the product within 16 min, with a radiochemical yield of 49.2 ± 8.6% and a radiochemical purity of 97.8 ± 1.0% determined by radio-thin layer chromatography. Starting with activities of 89 ± 14 GBq, molar activities were 290.9 ± 62.1 GBq/µmol, which is lower than for [^18^F]SiTATE, but can be attributed to the high precursor amount used in the synthesis.

## Pitfalls in SiFA automation

Most problems encountered using SiFA chemistry can be attributed to the eluent cocktail. Aqueous solutions of KOH and K_222_ are mixed at the desired ratio and lyophilized to dryness. Overnight drying is sufficient with a standard benchtop apparatus with a condenser temperature of − 50 °C, at a vacuum of approx. 125 mbar. The complex can be stored in a freezer for up to 1 year. Upon dissolution in acetonitrile, the complex has to be used quickly as the elution efficiency drops with time. It is recommended to use it within 15 min after dissolution and to mount the eluent vial on the cassette in the very last step before activity is delivered and the synthesis is started. A light yellowish color may occur, which does not affect the elution capability. Complete discoloration however indicates the decomposition of the complex. An in situ preparation of the complex by dissolving K_222_ and KOH in acetonitrile does not work.

The amount of oxalic acid has to be optimized for every individual automation setup, to achieve optimal radiochemical conversion. The amount of acid has to be precisely adjusted to balance the amount of base from the elution cocktail. This largely depends on the equipment used in the setup, such as length of the tubing and dead volumes within the ASU. 50 nmol precursor has shown to be sufficient to achieve a good conversion even in large batch productions. With lower precursor amounts, the radiochemical conversion might drop when high starting activities are used.

It is important to stabilize the product with ascorbate to prevent radiolytic degradation, since the radioactivity concentration can be extremely high on the cartridge during workup and in the product solution after elution from the cartridge. The formulation solution should be slightly acidic to prevent hydrolysis. At basic pH, [^18^F]fluoride will be released over time compromizing radiochemical purity. [^18^F]SiTATE is prone to stick to certain plastic materials. To reduce unspecific adsorption within vials, capillaries and syringes, Tween 20 can be added to ensure solubility. Tween 20 is a non-toxic polysorbate-type surfactant used in many pharmacological and food applications (Jones et al. 2018) and has shown to effectively reduce radioactive residuals on plastic walls facilitating clinical application.

## Quality control of SiFA radiopharmaceuticals

To release SiFA radiopharmaceuticals for clinical application, it is of utmost importance to make sure that specific quality criteria are met. Thus, suitable quality control measures following the manufacturing process are mandatory. The Ph. Eur. (European Pharmacopoeia) does not contain monographs covering SiFA tagged radiopharmaceuticals. However, the monographs “PSMA-1007 (^18^F) injection” (Ph. Eur. 10.5/3116) as well as “(^68^Ga) Galliumedotreotide solution for injection” (Ph. Eur. 10.6/2482) and “Gallium (^68^Ga) PSMA-11 injection” (In Ph. Eur. 10.4/3044) provide some orientation applicable to [^18^F]SiTATE and [^18^F]Ga-rhPSMA-7/-7.3 quality control analyses. In addition, there are also IAEA (International Atomic Energy Agency) guidelines published in the TECDOC series No 1856—“Quality Control in the Production of Radiopharmaceuticals” which give recommendations for quality control processes for radiopharmaceuticals intended for human use (IAEA 2018).

From these sources, it is possible to compile specifications and appropriate test methods, which have to be adapted according to the technical and instrumental preconditions of the respective production site and according to the regulatory standards of local authorities. The specifications for [^18^F]SiTATE that are used at LMU Munich and results obtained from 308 routine productions are summarized in Table 1.

**Table 1:** Specifications and QC results for [^18^F]SiTATE manufactured at LMU Munich (n = 308)

Test	Specifications	Results	Method
Appearance	Clear, colorless, no particles	Pass	Visual inspection
Radiochemical purity	≥ 95%	97 ± 0.9%	HPLC^a^
Chemical purity	≤ 5.7 µg/ml	2.1 ± 1.6 µg/ml	HPLC^a^
Identity	0.93–1.07 RRT^b^	0.97 ± 0.01	HPLC^a^
Residual solvents	≤ 10% (v/v) EtOH ≤ 410 ppm MeCN	5.8 ± 0.7% 29 ± 6 ppm	GC^c^
Half life	99–120 min	110 ± 1.32 min	Dose calibrator
Radionuclide identity	511 keV	Pass	Gamma spectrometry
Radionuclide purity	≥ 99.9%	Pass	Gamma spectrometry
pH	5–7	6.2 ± 0.2	pH meter
Kryptofix K_222_	≤ 733 µg/ml	Pass	Colorimetric spot test
Endotoxins	≤ 10 EU/ml	Pass	LAL test^d^
Sterility	Sterile	Sterile	Membrane filtration
Filter integrity	≥ 3.3 bar	Pass	Bubble point test

^a^High performance liquid chromatography

^b^Relative retention time

^c^Gas chromatography

^d^Limulus amebocyte lysate

## Quality control equipment and methodologies

To verify if the appearance of the product meets the release criteria, visual inspection is recommended. Color changes may be determined by holding the transparent glass vial in front of a color chart. [^18^F]SiTATE preparation is usually colorless which can best be checked in front of a white background. Light yellowish color, which results from formulation additives such as ascorbic acid, might appear and is also acceptable. The presence of any particulate matter is checked by gentle shaking of the QC vial. Any turbidity or particles have to be absent.

The radiochemical purity and identity of the products is determined by ultra- or high-performance liquid chromatography (UPLC/HPLC) with a suitable radioactivity and UV detector. Good separation is achieved e.g. for [^18^F]Ga-rhPSMA-7/-7.3 on a Nucleosil 100-5 C18 column (4 × 125 mm), solvent gradient with (A) water + 0.1% trifluoro acetic acid (TFA) and (B) acetonitrile: 30–38% (B) in 9 min, 38–95% (B) in 8 min, back to 30% (B) in 1 min and re-equilibration at 30% (B) for 1.5 min. ABX recommends similar conditions for the analysis of [^18^F]SiTATE using an Ascentis Express Peptide ES-C18 (4.6 × 150 mm) column and a solvent gradient with (A) Water + 0.1% TFA and (B) acetonitrile + 0.1% TFA: 40% (B) for 5 min, 40–95% (B) in 5 min, 95% (B) for 7 min, back to 40% (B) in 3 min. It is recommended to run a system suitability test prior to the analysis to ensure that the system is ready for analysis and well equilibrated. This test should at least include a blank run and a test run of the calibration standard. The identity of the radiotracer is determined by comparison of the retention times of the product and the non-radioactive standard. Radiochemical purity is calculated by integration of all relevant signals in the chromatogram and determination of the respective peak area ratio. It should be ensured that the fluorine-18 is completely eluted through the reversed-phase HPLC column. Insufficient elution leads to underestimation of potential fluorine-18 residuals, which in turn results in a false high radiochemical purity of the product. Polymeric columns are recommended, or when silica based C18 columns are used, a mobile phase with pH > 5 is preferred (Ory et al. 2015). The recovery of fluorine-18 should be determined during the method validation in advance. Alternatively, the fluorine-18 content can be reliably determined via thin layer chromatography (TLC) on silica gel coated TLC sheets using e.g. 50% aqueous acetonitrile as mobile phase for [^18^F]SiTATE or a 3:2 mixture (v/v) of acetonitrile in water, supplemented with 10% 2 M aqueous sodium acetate solution and 1% TFA for [^18^F]Ga-rhPSMA-7/-7.3, respectively. In this case, a radioactivity TLC scanner is needed to determine the radioactivity distribution. The radiochemical purity is defined as the ratio of counts in the product peak compared to total counts on the TLC plate. The experience shows that the radiochemical purity of the product is largely dependent on the chemical purity of the cold SiTATE starting material. Any SiFA containing contaminants will be also labeled just as the precursor, so that they will potentially deteriorate the final radiochemical purity of the product. Hence, a high precursor purity is considered essential.

The chemical purity can be assessed in different ways. By default, the peak area in the UV trace of the HPLC chromatogram corresponding to the product is determined. Using a calibration equation from a series of injections of an external standard with known concentrations, the chemical purity can be calculated. One-point calibration using a single injection of an external standard with known concentration is generally accepted as well. SiFA chemistry normally proceeds very cleanly and is devoid of by-product formation. However, all impurities, if any, have to be identified and quantified using a reference standard. Given that the precursor and the final product are chemically identical, except for the incorporated isotope (^19^F vs ^18^F), and cannot be separated, it can be assumed that the amount of SiFA precursor is the same as the radiolabeled [^18^F]SiFA product. This provides a simple and fast estimate to calculate the specific or molar activity of the final product. Residual solvents are quantified by gas chromatography. Ethanol is an integral part of the synthesis and designated as excipient in the product formulation. Up to 10% (v/v) ethanol content is accepted, whereas lower limits exist for other solvents. Small amounts of acetonitrile are common, which result from the labeling reaction, but must not exceed 410 ppm.

Radionuclidic identity is determined via gamma spectrometry. Fluorine-18 decays by positron emission forming two 511 keV photons upon annihilation with an electron. The photons interact with the detector material giving the characteristic signal in the gamma spectrum. However, this is true for all positron emitters. Identity has to be confirmed by measurement of the characteristic half-life of fluorine-18 in a dose calibrator. To assess radionuclidic purity, a retained sample is measured after 3 days again in a gamma spectrometer to identify any long-lived radionuclides. Peaks are quantified and summed up. The activity assigned to long-lived impurities is put in relation to the total activity end-of-synthesis. Due to the standardized and well-established production of fluorine-18 in the cyclotron, radionuclidic impurities are rarely found and usually below the legal limits.

The pH of the product formulation can be determined using a calibrated glass electrode pH meter or pH indicator strips. The solution should be slightly acidic to prevent hydrolysis of the SiFA moiety to the corresponding silanol under basic conditions.

Kryptofix 222 (K_222_) is a potentially harmful chemical, and the K_222_ content has to be controlled. Ph. Eur. monographs of ^18^F-labeled radiopharmaceuticals prescribe a threshold value of 2.2 mg/V. V designates the maximum injectable volume. To assess K_222_ concentrations, a simple color spot test can be used. A standard solution with the limit concentration is used as reference and iodine as dyeing agent. K_222_ contents are significantly higher if the cartridge based drying method is applied compared to the azeotropic drying technique.

To characterize the final formulation completely, the determination of the ascorbic acid content may be required. This can be done by iodometric analysis. A starch solution is used as indicator forming a blue complex in the presence of iodine. If the ascorbic acid content exceeds the test concentration predefined by the respective iodine content, the blue solution turns colorless.

Furthermore, quality control includes a test on bacterial endotoxins originating from gram-negative bacteria. This is achieved using commercially available test cartridges utilizing the reaction of endotoxins with the limulus amebocyte lysate extract from the horseshoe crab based on a chromogenic kinetic analysis method. Details can be taken from the monograph Ph.Eur. 10.0/2.6.14. Common methods for sterility testing are membrane filtration or direct inoculation. Sterility testing can be outsourced to external service providers since most manufacturers do not have the capacity and resources to make the necessary effort. Since incubation of the test samples in nutrient media takes 14 days, the results will be obtained retrospectively. It is imperative that no microbial growth is tolerated. To make sure that sterility is maintained, the dispensing should be done in a clean room environment under class A conditions including sterile filtration. Filter integrity is commonly checked by applying a bubble point test after sterile filtration, but before the administration of the radiopharmaceutical to the patient. In addition to quality control measurements, an extensive online monitoring of particles, pressure levels and laminar airflows during the dispensing of the product is mandatory.

## [^18^F]SiTATE stability

Two factors influence the stability of ^18^F-labeled SiFA radiopharmaceuticals, namely radiolytic degradation and hydrolysis under basic conditions. SiFA tagged radiotracers can be manufactured in high-dose productions. For example, at LMU, radioactivity concentrations of up to 2500 MBq/mL were achieved. Especially on cartridges, where the radiotracer is retained in a very small volume, radiolytic processes may easily occur. Ethanol itself, required to elute the product from the purification cartridge, has some radical scavenger capacities. However, it is advantageous to add further stabilizers such as ascorbic acid to the product to prevent radiolysis. It is also important that the formulation buffer is slightly acidic. It is recommended to adjust the pH of the formulation buffer to 6 by the addition of phosphoric acid. Under basic conditions, partial hydrolysis and a release of [^18^F]fluoride is found over time. A slightly acidic pH in the final formulation and the addition of ascorbic acid as stabilizer effectively prevents radiolytic and hydrolytic degradation.

## Conclusions and future perspectives

The SiFA radiochemistry has successfully made its entry into clinical radiotracer application demonstrating a high tracer production reliability, a lack of adverse patient reaction after injection and an unprecedented cost efficiency for large batch production. The first two radiotracers for the diagnosis of neuroendocrine tumors and prostate cancer, [^18^F]SiTATE and [^18^F]Ga-rhPSMA-7/-7, and their application in a large cohort of patients have consolidated the clinical value of the SiFA methodology. Manufacturers of ^18^F-radiopharmaceuticals already caught attention of SiFA chemistry, starting to implement SiFA precursor syntheses and SiFA radiotracer development into their commercial portfolio. These are all signs that SiFA has found its niche within the vast landscape of radiopharmacy. Whether SiFA radiopharmaceuticals can consolidate a permanent position among clinically used radiotracer remains to be seen and will be crucially dependent on careful cost–benefit calculations in comparison to other radionuclide-based imaging agents. Undeniably, the use of fluorine-18 is advantageous in diagnostic PET imaging, providing a strong incentive to optimize kit-like radiochemistries such as SiFA.

## Data Availability

All data generated or analysed during this study are included in this published article.

## Abbreviations

SiFA: Silicon fluoride acceptor
PET: Positron emission tomography
A_m_: Molar activity
ASU: Automated synthesis unit
GMP: Good manufacturing practice
TATE: (Tyr^3^)-octreotate
PSMA: Prostate-specific membrane antigen
IE: Isotopic exchange
PBS: Phosphate buffered saline
DMSO: Dimethylsulfoxide
KOH: Potassium hydroxide
K_222_: Kryptofix® 222
RP: Reversed phase
GEP-NET: Gastroenteropancreatic neuroendocrine tumor
QC: Quality control
HPLC: High-performance liquid chromatography
Ph. Eur.: European Pharmacopoeia
IAEA: International Atomic Energy Agency
RRT: Relative retention time
GC: Gas chromatography
LAL: Limulus amebocyte lysate
TFA: Trifluoro acetic acid
TLC: Thin layer chromatography
